# Awareness and acceptability of malaria vaccine among caregivers of under-5 children in Northern Nigeria

**DOI:** 10.1186/s12936-023-04768-z

**Published:** 2023-10-31

**Authors:** Mary Yetunde Ajayi, Daniel Chukwuyere Emeto

**Affiliations:** 1 Department of Epidemiology and Medical Statistics, Faculty of Public Health, College of Medicine, University of Ibadan, Ibadan, Nigeria; 2 Leaders of Africa Institute, Accra, Ghana

**Keywords:** Malaria, Vaccine, Awareness, Acceptability, Under-5, Caregivers

## Abstract

**Background:**

Malaria vaccine, RTS, S/AS01, has demonstrated modest efficacy against malaria and holds promises for children living in areas where malaria transmission is high. This study assessed caregivers’ awareness and willingness to accept the vaccine and provided vital information for policymakers, health workers, and social mobilizers on critical areas to focus on promoting the new vaccine uptake before its arrival for use in Nigeria.

**Methods:**

The study was a community-based cross-sectional survey. A multistage sampling technique was used to select four states in Northern Nigeria, where the incidence and prevalence of malaria are highest in the country and 504 caregivers of under-five children were interviewed. A semi-structured interviewer-administered questionnaire was used and data analysis was done with p-value at 0.05.

**Results:**

Two hundred and three (40.3%) respondents were aware of malaria vaccine while four hundred and sixty-three (91.9%) were willing to accept the vaccine when it is introduced for use in the country. Level of education (AOR; 0.42; CI 0.23–0.78), employment status (AOR; 3.03; CI 1.82–5.03), previous experience of other childhood vaccinations (AOR; 4.87; CI 2.89–8.20), and caregivers having suffered malaria within the last one year (AOR; 1.85; CI 1.10 −3.13) significantly predicted malaria vaccine awareness. Awareness of the new malaria vaccine (AOR = 6.88; 95% CI 1.53–30.99), and previous experience of other childhood vaccinations (AOR = 6.16; 95% CI 2.54–14.94) were significant predictors of the vaccine acceptability.

**Conclusion:**

Caregiver’s awareness of the new malaria vaccine was inadequate. There is a need to intensify efforts on social and behavioural communication change activities tailoring messages on the vaccine to address uptake hesitancy. Also, an intense community engagement with focus on providing information on the safety of the vaccine is encouraged.

## Background

Several approaches including indoor residual spray, insecticide-treated nets, chemoprevention, environmental modification, and malaria case management have been used to reduce the prevalence and impact of malaria disease for decades [1, 2]. Despite these interventions, malaria remains a primary cause of childhood illness and death in the World Health Organization (WHO) African Region. In 2021, 247 million malaria cases and 619,000 estimated malaria deaths were reported worldwide [3]. The African region bears 95% and 96% of the cases and death, respectively, and under-5 children bears 80% of malaria death in the region [3]. Nigeria (31.3%), the Democratic Republic of Congo (12.6%), the United Republic of Tanzania (4.1%), and Niger (3.9%) bear more than half of the burden of malaria worldwide [3].

In the pursuit for new and more effective malaria control modalities, the malaria vaccine RTS,S/AS01 (RTS,S) was tested in a large-scale phase 3 clinical trial between 2009 and 2014 in moderate to high malaria transmission regions, the results showed that malaria cases dropped by more than half within 12 months and by 40% after four years of follow up [3, 4]. In 2019, a pilot implementation of the vaccine was carried out in Ghana, Kenya, and Malawi and as of 2022, about 1.5 million children at risk of the disease have been vaccinated across the three African Countries [3]. Findings from the pilot programme showed that RTS,S is safe and effective for use and significantly reduced the incidence of severe malaria cases and child death due to malaria [3]. Thus, the WHO recommended the use of the RTS, S malaria vaccine for the prevention of *Plasmodium falciparum* malaria in children living in regions with moderate to high transmission [3, 4]. RTS,S is the first recommended vaccine for malaria and any parasitic disease. It acts against *P. falciparum*, the deadliest malaria parasite globally and the most prevalent in sub-Saharan Africa [3, 4].

The use of vaccines has been successfully demonstrated in the fight against several infectious diseases like measles, polio, smallpox, tetanus, tuberculosis, and meningitis. Hence, the introduction of the malaria vaccine will represent an important achievement in the control and elimination of the disease [5]. There are strong indications that a second malaria vaccine, R21/Matrix-M (R21), might be introduced in other countries, including Nigeria, by 2024 [6]. This will be a major milestone and if implemented without hindrance could save tens of thousands of lives. However, previous introduction of vaccination programmes in Nigeria has experienced difficulties including awareness, trust, and acceptability which could pose a threat to a successful administration of malaria vaccine in the country [7].

The intensity and seasonality of malaria transmission varies considerably across the highly diverse ecological zones in Nigeria. The risk of transmission exists throughout the country. However, the incidence and prevalence of malaria is highest in the northern and north-eastern parts of the country [20]. Thus, with the initiation of the High Burden High Impact programme for malaria control, the deployment of a malaria vaccine intervention may commence in this region due to its epidemiology, as has been done for other malaria prevention interventions for under 5 children, such as the Seasonal Malaria Chemoprevention [21, 22]. Hence, this study assessed the awareness and acceptability of the malaria vaccine among caregivers of under-5 children in Northern Nigeria and identified factors influencing their awareness and acceptability to prepare and develop remedial action before its arrival and administration in Nigeria.

## Methods

### Study area

This study was done in Northern Nigeria. Northern Nigeria is made up of three geopolitical zones including North Central, Northeast, and Northwest. The region is composed of 19 states including the Federal Capital Territory. Northern Nigeria is the largest region in Nigeria and makes up over 70% of the land mass of the country with a population of over 75 million people. Islam is the dominant religion in the area and Hausa and Fulani are the predominantly spoken languages.

### Study design

A community-based cross-sectional study design was employed for this study.

### Study population

The study population was caregivers of under 5 children.

### Inclusion and exclusion criteria

The study included caregivers of under-5 children who gave their consent to be part of the study while caregivers who do not give consent, ill, and physically unfit were excluded from the study.

### Sample size determination

The required sample size for this study was estimated using the Leslie Kish formula for single proportion assuming a 95% level of confidence, proportion of respondents aware of malaria vaccine at 18% [17]; 5% precision and 10% for anticipated non-response rate. This gives a minimum sample size of 252 per group.

### Sampling technique

A multistage sampling procedure was used to recruit respondents. Four states were selected from Northern Nigeria at least one from each geopolitical zone in the region using simple random sampling. The sampling frame of all the LGAs in each selected state was obtained and two LGAs was selected from each state (one rural LGA and one urban LGA each) using simple random sampling. Sixty-three caregivers of under-five children were enrolled as respondents in the study from each of the selected LGAs by balloting.

### Study instrument

A pretested semi-structured, interviewer-administered, forty-item questionnaire was used to elicit information from respondents. The questionnaire was divided into four sections, namely (i) Respondents' socio-demographic characteristics, including age, sex, occupation, and marital status; (ii) Health-related characteristics of caregivers and child, including having an incidence of malaria in the last one year; (iii) Previous experience of childhood vaccination; and (iv) Caregivers awareness and acceptability of malaria vaccines.

### Data collection

A total of 504 caregivers of under-five children participated in the study. The questionnaire was administered by trained Research Assistants in the respondent’s respective homes. The data was collected between February and March 2023.

### Data management/analysis

STATA was used to analyse the data obtained. Descriptive statistics were computed using summary statistics such as frequency, mean, percentages, and standard deviations. The relationship between the independent variables and dependent variables was assessed using binary logistic regression. To control potential confounders, all significant variables were considered for multivariable logistic regression which was applied to determine the independent effect of each variable on the outcome variables.

## Results

### Socio-demographic characteristics of respondents

A total of 504 caregivers of under 5 children in northern Nigeria participated in the study. The mean age of caregivers was 33.5 ± 8.5 years, and the majority of the respondents (45.6%) were in the 30–39 years age group. Most of the respondents were female (82.1%), married (89.3%), and of the Islamic faith (59.9%). One hundred and ninety-two (38.1%) of the caregivers had completed tertiary education at the time of the study, 303 (60.1%) were employed and 42.7% earned between 193.69 USD and 645.63 USD annually (Table 1).

**Table 1:** Socio-demographic characteristics of survey respondents

Variables	n (%)
Age group (years)	
18–29	171 (33.9)
30–39	230 (45.6)
40–49	72 (14.3)
≥ 50	31 (6.2)
Location	
Urban	252 (50.0)
Rural	252 (50.0)
Gender	
Female	414 (82.1)
Male	90 (17.9)
Marital Status	
Married	450 (89.3)
Others	39 (7.7)
Single	15 (3.0)
Religion	
Islam	302 (59.9)
Christianity	202 (40.1)
Level of Education	
No formal schooling	106 (21.0)
Primary	46 (9.1)
Secondary	160 (31.8)
Tertiary	192 (38.1)
Employed	
No	201 (39.9)
Yes	303 (60.1)
Occupation	
Unemployed	27 (5.4)
Self-employed	194 (38.5)
Formally employed	115 (22.8)
Housewife	124 (24.6)
Others	44 (8.7)
Family size	
< 5	185 (36.7)
5–10	280 (55.6)
> 10	39 (7.7)
Number of under-5 children	
1	239 (47.4)
≥ 2	265 (52.6)
Household Annual Income (USD)^a^	
< 193.69/year	111 (22.0)
193.69–645.63/year	215 (42.7)
> 645.63/year	178 (35.3)

Mean Age = 33.5 ± 8.5 with a minimum age of 18 and maximum age of 66

Median Age with Inter Quartile Range (IQR) = 32 (10)

Mean Family size = 5.9 ± 3.2 with a minimum of 2 and maximum of 27

Mean Number of under-5 children = 1.6 ± 0.7 with a minimum of 1 and maximum of 6

^a^Conversion rate used was 774.44 naira to 1 USD and the source was: https://www.cbn.gov.ng/rates/exchratebycurrency.asp

### Health-related characteristics

More than half, 374 (74.2%) of the caregivers and 450 (89.3%) of those under-5 years had malaria in the past year.

### Previous experience with childhood vaccination

The majority of the caregivers were conversant with the BCG vaccine (91.1%), followed by hepatitis B (81.4%), OPV (88.3%), and pentavalent (86.1%). Other vaccines included pneumococcal conjugate (80.2%), rotavirus (80.8%), inactivated polio vaccine 410 (81.3%), and measles 419 (83.1%). (Table 2).

**Table 2:** Caregivers’ with previous experience with childhood vaccination

Variables*	n (%)
BCG	459 (91.1)
Oral polio (OPV)	445 (88.3)
Pentavalent vaccine (PENTA)	434 (86.1)
Vitamin A	427 (84.7)
Measles	419 (83.1)
Hepatitis B	410 (81.4)
Inactivated polio vaccine (IPV)	410 (81.3)
Rotavirus (ROTA)	407 (80.8)
Pneumococcal conjugate (PCV)	404 (80.2)
Yellow fever	375 (74.4)
Meningitis	366 (72.6)
Cholera	269 (53.3)
Typhoid fever	266 (52.8)
Chickenpox	248 (49.2)

^*^ = multiple responses

### Awareness of the malaria vaccine

Only 203 (40.3%) of the caregivers have heard about the malaria vaccine. Of those who have heard one hundred and sixty-seven (82.3%) got the information from healthcare provider, family/friends 99 (48.8%), and media (TV, radio) 37 (18.2%). Two hundred and sixty-two (52.0%) knew the eligible recipients of the malaria vaccine (Table 3).

**Table 3:** Awareness of malaria vaccine among caregivers

Variables	n (%)
Ever heard about the malaria vaccine	
No	301 (59.7)
Yes	203 (40.3)
Source of Information*	
Healthcare provider	167 (82.3)
Media (TV, radio)	37 (18.2)
Family or friends	99 (48.8)
Religious leaders	8 (3.9)
Government agencies	15 (7.4)
Social media	22 (10.8)
Know who is eligible to receive the malaria vaccine	
No	242 (48.0)
Yes	262 (52.0)

^*^ = multiple responses

### Willingness to accept malaria vaccine

Four hundred and sixty-three (91.9%) of the caregivers were willing to accept the malaria vaccine for their child when it is available. The main reasons for unwillingness to vaccinate their child were fear of adverse reaction (56.2%) and spousal refusal (26.8%) while the main reasons for willingness were to protect the child (64.4%) and general vaccine acceptance (16.6%). The majority 452 (89.7%) were willing to pay to receive the malaria vaccine (Table 4).

**Table 4:** Willingness to accept malaria vaccine

Variables	n (%)
Willingness to accept malaria vaccine	
No	41 (8.1)
Yes	463 (91.9)
Reasons for unwillingness	
Fear of adverse reaction	23 (56.2)
Spousal refusal	11 (26.8)
Cultural belief	3 (7.3)
Others (Not interested)	2 (4.9)
Religious belief	1 (2.4)
Cost of vaccine	1 (2.4)
Reasons for willingness	
Protect the child	298 (64.4)
General vaccine acceptance	77 (16.6)
Accepting but safety concerns	45 (9.7)
Protect others	37 (8.0)
Prior experience with childhood vaccination	6 (1.3)
Willingness to pay for malaria vaccine	
No	52 (10.3)
Yes	452 (89.7)

### Factors associated with awareness of the malaria vaccine

Only 203 (40.3%) of the total respondents were aware of the malaria vaccine. Education level, employment status, previous experience with childhood vaccinations and caregivers who had malaria in the last year were all significant predictors of awareness of the malaria vaccine. The odds of caregivers with secondary education as their highest level of education being aware of the malaria vaccine was 58% less than caregivers without formal education (AOR; 0.42; CI 0.23 −0.78) while the odds of caregivers with tertiary education as their highest level of education being aware of the malaria vaccine was 51% less than caregivers without formal education (AOR; 0.49; CI 0.26 −0.93). Employed caregivers were 3.0 times more likely than unemployed caregivers to be aware of the malaria vaccine (AOR; 3.03; CI 1.82 -5.03).

Caregivers who had previous experience with childhood vaccinations were 4.9 times more likely to be aware of the malaria vaccine than their counterparts without prior experience with childhood vaccinations (AOR; 4.87; CI 2.89 −8.20). Also, caregivers who had malaria in the last year were 1.9 times more likely to be aware of the malaria vaccine than caregivers who didn’t have malaria in the last year (AOR; 1.85; CI; 1.10 −3.13) (Table 5).

**Table 5:** Factors associated with awareness of the malaria vaccine

Variables	COR(95% CI)	P-value	AOR (95% CI)	P-value
Age group				
18–29				
30–39	1.41(0.94–2.12)	0.09		
40–49	1.09(0.62–1.92)	0.76		
≥ 50	0.60(0.25–1.41)	0.24		
Location				
Rural				
Urban	1.20(0.84–1.71)	0.32		
Gender				
Female				
Male	0.51(0.31–0.84)	0.01*		
Marital status				
Married				
Single	0.53(0.17–1.69)	0.28		
Others	1.01(0.52–1.97)	0.97		
Religion				
Christianity				
Islam	1.05(0.73–1.51)	0.80		
Level of Education				
No formal schooling				
Primary	1.36(0.68–2.71)	0.39	0.96(0.43–2.17)	0.93
Secondary	0.60(0.36–1.00)	0.05*	0.42(0.23–0.78)	0.01*
Tertiary	1.10(0.68–1.78)	0.70	0.49(0.26–0.93)	0.03*
Employed				
No				
Yes	2.44(1.67–3.57)	0.00*	3.03(1.82–5.03)	0.00*
Family size				
< 5				
5–10	1.26(0.86–1.84)	0.24		
> 10	0.48(0.22–1.07)	0.07		
Number of under-5 children				
1				
≥ 2	1.36(0.95–1.95)	0.09		
Previous experience with childhood vaccinations				
No				
Yes	6.07(3.74–9.83)	0.00*	4.87(2.89–8.20)	0.00*
Caregiver had malaria in the last 1 year				
No				
Yes	2.44(1.57–3.82)	0.00*	1.85(1.10–3.13)	0.02*
Child had malaria in the last 1 year				
No				
Yes	3.30(1.62–6.73)	0.00*		

^*^Significant p-value

### Factors associated with the acceptability of the malaria vaccine

Health-related characteristics, previous experience with childhood vaccination, awareness of the malaria vaccine and socio-demographic characteristics were used to run bivariate logistic regression. Statistically significant variables were considered for multivariable logistic regression. Awareness of the malaria vaccine and previous experience with childhood vaccinations were found to be significantly associated with willingness to accept the malaria vaccine at a p-value ≤ 0.05. Caregivers of under-5 children who were aware of the malaria vaccine were 6.9 times more likely willing to vaccinate their child compared to those who were not aware of the vaccine (AOR = 6.88; 95% CI 1.53–30.99). Caregivers with prior experience with childhood vaccination were 6.2 times more likely than caregivers with no prior experience to vaccinate their child (AOR = 6.16; 95% CI 2.54–14.94) (Table 6).

**Table 6:** Factors associated with the acceptability of the malaria vaccine

Variables	COR(95% CI)	P-value	AOR (95% CI)	P-value
Age group				
18–29				
30–39	0.73(0.33–1.63)	0.44		
40–49	0.68(0.24–1.96)	0.48		
≥ 50	0.21(0.07–0.61	0.00*		
Location				
Rural				
Urban	1.31(0.69–2.48)	0.42		
Gender				
Female				
Male	0.56(0.27–1.17)	0.12		
Marital status				
Married				
Single	1.29(0.17–10.09)	0.81		
Others	1.71(0.40–7.36)	0.47		
Religion				
Christianity				
Islam	0.46(0.22–0.95)	0.04*		
Education				
No formal schooling				
Primary	2.55(0.70–9.22)	0.15		
Secondary	1.85(0.86–3.98)	0.11		
Tertiary	4.09(1.69–9.91)	0.002*		
Employed				
No				
Yes	2.28(1.19–4.37)	0.01*		
Family size				
< 5				
5–10	0.82(0.38–1.76)	0.61		
> 10	0.18(0.07–0.47)	0.000*		
Number of under-5 children				
1				
≥ 2	1.06(0.56–2.01)	0.86		
Awareness of the malaria vaccine				
No				
Yes	14.96(3.57–62.69)	0.000*	6.88(1.53–30.99)	0.01*
Previous experience with childhood vaccinations				
No				
Yes	11.03(4.96–24.53)	0.000*	6.16(2.54–14.94)	0.000*
Caregiver had malaria in the last 1 year				
No				
Yes	1.55(0.79–3.06)	0.21		
Child had malaria in the last 1 year				
No				
Yes	3.07(1.41–6.68)	0.01*		

^*^significant p-value

## Discussion

The RTS,S malaria vaccine has demonstrated modest efficacy against malaria and it is promising for children living in regions where malaria transmission is high. Understanding caregivers’ level of awareness and willingness to accept the vaccine is vital to inform policy makers, health workers, and social mobilizers on critical areas to intensify efforts on promoting the vaccine uptake before its approval for use in Nigeria [23].

Nigeria bears the largest burden of malaria worldwide [8], with children under-five most severely affected [9]. This study revealed that almost all the under-5 children (89.3%) of the respondents had malaria within the last one year prior to the survey. This calls for the need for the regulatory authorities, e.g. NAFDAC, to expedite action in the approval of the vaccine for use in the country.

The awareness level of the malaria vaccine among caregivers of under-5 children in this study is poor. Similar reports of low malaria vaccine awareness in the country were reported by Abdulkadir and Ajayi in 2015 among caregivers of under-5 children in Oyo State, Chukwuocha et al*.* who accessed the awareness and perception to comply with prospective malaria vaccine in Enugu State in 2018 and Musa-Booth et al*.* study in Abuja among mothers in 2021 [2, 10, 11]. Thus, given Nigeria’s prevalence of malaria among under-5 children, it is increasingly likely that the vaccine will be introduced in the country, therefore, there is a need to intensify efforts on Social and Behaviour Change Communication (SBCC) activities tailoring messages on the vaccine to avoid or limit uptake hesitancy. The major source of information among the caregivers was from healthcare providers. This could be because caregivers trust health professionals and rely on them for verifiable health information [12]. Integrating messages of the prospective arrival of malaria vaccine alongside current malaria interventions like the long-lasting insecticidal nets (LLINs), Seasonal Malaria Chemoprevention, and others been currently implemented and championed by healthcare workers at the community and facility levels should be considered.

Previous experience with childhood vaccination was significantly associated with awareness of the malaria vaccine. It was encouraging to see that more than half of the caregivers had a good knowledge of childhood vaccination. This knowledge may be due to intensified and long-standing campaigns promoting childhood vaccination [10]. There is a need to also leverage this existing structure in promoting the malaria vaccine to boost awareness among caregivers. The association between childhood vaccination and malaria vaccine awareness may also be due to the fact that caregivers who had knowledge of child vaccination and take their children for vaccination have access to information on health including new vaccines from healthcare providers. This is corroborated by a study to access community perceptions of a malaria vaccine in the Kintampo districts of Ghana by Febir et al*.* [13] who reported that respondents with high knowledge of routine childhood vaccinations sought information on how their children could be vaccinated against all diseases including malaria.

Another significant factor associated with malaria vaccine awareness was the incidence of malaria among the caregivers within the past year prior to this study. Caregivers who had suffered malaria were more aware of the malaria vaccine than those who did not report having malaria within the same period. This may be because individuals who suffer from illness tend to source with intent health-related information due to their need for curative or preventive healthcare services for their illness [14]. Hence, there is a need to saturate all communication mediums, including social media, and the internet with messages on the prospective malaria vaccine prior to its arrival. Other factors found to be significantly associated with awareness were respondents’ level of education and employment status.

This study found that caregivers’ willingness to accept the malaria vaccine when made available in the country was very high. The main reason caregivers were willing to vaccinate their child with the malaria vaccine was the belief that it will help to protect the child. This belief may be due to successes recorded in other childhood vaccination programs like polio vaccination [10, 13]. A similar report of good acceptability was reported in other African countries including Ghana, Mozambique, and Kenya [13, 15, 16]. Furthermore, awareness of the malaria vaccine from this study was strongly associated with its acceptability, caregivers who were aware of the vaccine were 6.9 times more likely to vaccinate their child when the vaccine is introduced in the country. This finding corroborates studies done by Asmare [17], Onyekachi et al. [9], and Abdulkadir and Ajayi [10].

The primary reasons for caregivers’ reluctance to accept the malaria vaccine were due to fear of adverse reactions and spousal refusal. Similar findings were reported by [10] in Oyo State Southwestern Nigeria, and [18] in a review of acceptability, availability, and feasibility of RTS, S/AS01 Malaria Vaccine in 2023 and attributed such fears to limited community engagement and issues with quality health care service delivery. There is, therefore, an urgent need to intensify efforts on community engagement, interacting with key community/traditional and religious leaders, addressing their concerns, and providing information on the safety of the vaccine. Creation of locally tailored content like videos and visuals of success stories from locals of countries where the vaccine has been successfully utilized to boost acceptance is recommended.

Although financial challenges remain a significant hindrance to accessing health care services and vaccine acceptability in sub-Saharan Africa [18], it was encouraging to see that the majority of the respondents were willing to pay for the malaria vaccine. This agrees with findings by Ojakaa et al. [16] and by Abdulkadir and Ajayi [10], but not with a study by Asmare [17] in Ethiopia where respondents reported that they might not be able to afford the vaccine and asked for the vaccine to be free of charge. Although most of the respondents would pay for the vaccine the price may not be affordable for many households [2], thus, the possibility of subsidizing the cost when introduced in the country is advised.

This study also compared key factors between urban and rural locations in the event that different approaches to effort intensification are required. However, the location did not play a significant role in caregivers’ willingness to accept RTS,S which is contrary to studies done in Kenya and Northwest Ethiopia [16, 19]. This could be attributed to increased utilization of health services through primary health centers (PHCs) established in both rural and urban areas of the country as the Nigerian government has increased efforts to ensure universal health coverage across the country through the use of community mobilization strategies, the provision of health amenities, and education. This study is limited in that awareness was measured quantitatively, asking respondents if they have heard of the vaccine, hence, subject to information bias. A more in-depth study using qualitative variables is recommended. Despite this, findings from the study are very useful as it will help inform programmes to ease the introduction of malaria vaccine in Nigeria.

## Conclusion

In conclusion, although awareness of the WHO-approved malaria vaccine (RTS,S) is not high in Northern Nigeria, caregivers were eager to acquire the vaccine for their under-5 children when made available. Fear of adverse reactions was one of the reasons cited for refusal to receive the vaccine, caregivers would only give their children the vaccine if it was proven to be safe with no side effects and efficacious. Acceptability of the malaria vaccination was found to be substantially related to vaccine awareness in this study, hence, to achieve optimal acceptability of the malaria vaccine in Nigeria, constant health education at all levels is required, with an emphasis on the vaccination’s benefits. The government should also leverage media houses, social media, and religious leaders to enlighten the masses on the malaria vaccine prior to its availability in Nigeria.

## Data Availability

The dataset used and analysed during the current study is available from the corresponding author on request.

## Abbreviations

LGA: Local Government Area
RTS,S: Mosquirix
WHO: World Health Organization
SBCC: Social and Behaviour Change Communication
LLIN: Long-Lasting Insecticidal Net
